# Measurement challenges and causes of incomplete results reporting of biomedical animal studies: Results from an interview study

**DOI:** 10.1371/journal.pone.0271976

**Published:** 2022-08-12

**Authors:** Till Bruckner, Susanne Wieschowski, Miriam Heider, Susanne Deutsch, Natascha Drude, Ulf Tölch, André Bleich, René Tolba, Daniel Strech

**Affiliations:** 1 QUEST Center for Responsible Research, Berlin Institute of Health at Charité –Universitätsmedizin, Berlin, Germany; 2 Institute for Ethics, History and Philosophy of Medicine, Hannover Medical School, Hannover, Germany; 3 Institute for Laboratory Animal Science, Hannover Medical School, Hannover, Germany; 4 Institute for Laboratory Animal Science, RWTH Aachen University, Faculty of Medicine, Aachen, Germany; Bavarian Health and Food Safety Authority: Bayerisches Landesamt fur Gesundheit und Lebensmittelsicherheit, GERMANY

## Abstract

**Background:**

Existing evidence indicates that a significant amount of biomedical research involving animals remains unpublished. At the same time, we lack standards for measuring the extent of results reporting in animal research. Publication rates may vary significantly depending on the level of measurement such as an entire animal study, individual experiments within a study, or the number of animals used.

**Methods:**

Drawing on semi-structured interviews with 18 experts and qualitative content analysis, we investigated challenges and opportunities for the measurement of incomplete reporting of biomedical animal research with specific reference to the German situation. We further investigate causes of incomplete reporting.

**Results:**

The in-depth expert interviews revealed several reasons for why incomplete reporting in animal research is difficult to measure at all levels under the current circumstances. While precise quantification based on regulatory approval documentation is feasible at the level of entire studies, measuring incomplete reporting at the more individual experiment and animal levels presents formidable challenges. Expert-interviews further identified six drivers of incomplete reporting of results in animal research. Four of these are well documented in other fields of research: a lack of incentives to report non-positive results, pressures to ‘deliver’ positive results, perceptions that some data do not add value, and commercial pressures. The fifth driver, reputational concerns, appears to be far more salient in animal research than in human clinical trials. The final driver, socio-political pressures, may be unique to the field.

**Discussion:**

Stakeholders in animal research should collaborate to develop a clear conceptualisation of complete reporting in animal research, facilitate valid measurements of the phenomenon, and develop incentives and rewards to overcome the causes for incomplete reporting.

## Introduction

The issue of incomplete reporting, when study outcomes are reported only partially or not at all, has attracted growing attention across numerous fields of natural and social science, raising questions about the rigour, efficiency, ethics and integrity of the scientific process, and the reliability, replicability and robustness of research findings [1–3].

In biomedical research, incomplete reporting and its effects on publication bias is well documented for human clinical trials [4, 5], but far less so for animal studies [6–9]. The lack of information about publication rates in animal research could be partly explained by practical barriers and conceptual uncertainties for the empirical measurement of publication rates. In principle, the measurement of incomplete reporting of results in biomedical research is facilitated by the requirement for researchers to obtain advance approval for studies involving humans or live animals. To obtain approval, researchers must pre-specify all planned experiments within a study and the number of ‘participants’ in each experiment. Reporting gaps in clinical trials have repeatedly been quantified by using ethics committee approvals or funder cohorts to establish a cohort of all studies conducted, and then searching the literature for their published outcomes [10–12]. In European Union member states, legally mandated approvals by official bodies could be used to establish similar cohorts of animal studies.

Two separate groups recently used this approach to quantify non-publication of animal research on the level of entire animal studies and on the level of approved animals numbers. One group, where several authors from this paper participated, found that of 158 approved studies at two German university medical centres that had verifiably been initiated, 33% had published outcomes neither in the scientific literature nor within doctoral theses [13]. Another group found that of 67 approved studies at a Dutch university, 40% did not result in publications [14]. But has the publication rate on the level of approved animal studies sufficient construct validity? This question is important because entire animal studies might reflect several experiments which all include different animals. A 2011 survey among 454 Dutch animal researchers asked for the publication rate on the experiment level. The surveyed researchers estimated that about 50% of all experiments remain unpublished [15]. Furthermore, the above mentioned follow-up study at a Dutch university also assessed the publication rate on the level of animal numbers mentioned in the 67 approved study applications and found that journal articles did not report outcomes for 74% of the mentioned animals.

What of these three different concepts for non-publication has the highest construct validity? The 33–40% at the *study* level [13, 14], the 50% at the *experiment* level [15], or the 74% at the *animal* level [14]? Furthermore, how good is the internal and external validity of these three types of measures? Because reporting on the number of animals used in specific experiments both in approval documents [16] and in journal publications [6] is limited the measurement of reporting rates at all three levels might face substantial challenges.

The goal of this study was to explore qualitatively the measurement challenges and causes of incomplete reporting of the results of animal studies, differentiating between three levels of measurement: (1) overall *study* as approved by regulators, (2) discrete *experiments* nested within the study, and (3) individual *animals* used within experiments.

## Methods

This study is reported in line with the Consolidated criteria for reporting qualitative research (COREQ) guideline.

We used purposive sampling to gain perspectives from different animal researchers and other stakeholders. The primary purpose was to obtain as complete a picture as possible of the different causes for incomplete results reporting in animal research and the opportunities and challenges in measuring incomplete reporting. Within the interviews we introduced three levels of analysis for results publication: study, experiment, and subjects. See Table 1 for examples and how to compare these three level of analysis with the area of clinical research.

**Table 1:** Incomplete publication of results: Three levels of analysis.

Level	Domain	Unit	Incomplete publication example
Study	CT	Entire clinical trial	Trial outcomes not reported at all
	***AS***	***Approved study***	***Study outcomes not reported at all***
Experiment	CT	Intervention arm or sub-group	Certain arms and/or sub-groups not reported
	***AS***	***Discrete experiment***	***Certain experiments not reported***
Subjects	CT	Individual patient	Non-reporting of dropouts, outliers, etc
	***AS***	***Individual animal***	***Non-reporting of outcomes for some animals***

Note: CT = human clinical trial, AS = biomedical animal study.

Due to the sensitivity of the subject matter, initial participants were recruited from the study team’s professional networks, with further participants recruited via snowballing. As our study team focuses on responsible research, this likely biased our sample towards respondents with a high awareness of issues relevant for incomplete reporting. Interviewees were offered 150 Euros to compensate them for the time required to participate in the study. Following a purposive and iterative sampling strategy we recruited 18 interviewees (from 26 contacted, response rate 69%) until we reached thematic saturation of mentioned topics. While respondents were drawn from multiple regions in Germany and multiple levels of seniority, ranging from postdoctoral researchers to senior academics, we did not attempt to recruit a representative but aimed for a purposive sample with diverse backgrounds and perspectives on the topic of comprehensive reporting. While governmental competencies might differ in some details across German federal states the overarching regulatory requirements and the concept of animal studies incorporating several animal experiments is the same throughout Germany. All interviewees met our inclusion criterion of having experience in conducting, supervising and/or publishing the findings of animal research (S1 File).

All participants were asked to sign a consent form that outlined the basic research questions, informed participants that interviews would be recorded and transcribed, and that their anonymity would be safeguarded. Participants consented to selected quotes from interviews being cited verbatim in a future publication (S2 File). All interviews were conducted via video call by the same member of the study team (TB), in 2 cases in conjunction with another team member (ND or UT), based on a written interview guide that had been developed by the team of authors in an iterative process (S3 File). Because the team of authors include five persons with background in animal research, we did not conduct pilot interviews. The lead interviewer is a German postdoctoral researcher with extensive experience in conducting qualitative research, including on publication bias, but with no personal experience of conducting animal research; his professional background was disclosed verbally at the outset of each interview. All interviews were between 50 and 70 minutes in length, with a mean length of 60 minutes. All interviews were transcribed by a professional transcription company that had signed a non-disclosure agreement.

The lead interviewer (TB) reviewed all transcripts and manually grouped responses into thematic categories initially broadly mirroring key items in the interview guide and subsequently further sub-categorising them until thematic saturation was reached as per the criteria elaborated by Fusch and Ness [17]. Further team members (SW, NT, UT, DS) reviewed and commented the categorisation; disputes were resolved by consensus. Quotes cited in the paper (as “Q99”) were selected by eliminating duplicate quotes on the same topic until arriving at the quote or quotes that best summarised the tenor of all responses received on that issue. Quotes were translated by a bilingual researcher (TB) and are available in German and English language (S4 File). The interview transcripts, slightly redacted to further safeguard the anonymity of participants, were archived on a password-protected server at the Charité, Berlin, Germany.

The study was preregistered on the Open Science Framework (https://osf.io/34qny/) and was approved by the Medizinische Hochschule Hannover, Hannover, Germany ethics committee (number 9504_BO_K_2020).

## Results

### Interviews

We conducted confidential semi-structured interviews with 18 experts (14 animal researchers, 2 methodology experts, 1 journal editor, 1 industry group representative; 16 located in Germany and 2 in UK) conducted during May-June 2021. The main categories identified in the interviews became increasingly saturated after approximately 10 interviews. While the next eight interviews provided further perspectives on sub-categories and particularities no new major categories emerged. While thematic saturation was reached for main categories we may not have captured minor or rare factors.

Quotes exemplifying the themes presented in the following sections are displayed in Table 2 and more exhaustively in S4 File.

**Table 2:** Selected quotes from interviewees.

MEASUREMENT OF INCOMPLETE REPORTING			
**Study level tracking challenges**			
Funding not secured	R07	Q1	For example with [German public funder] DFG you have to submit a [pre-] approved protocol (*Tierversuchsantrag)*. When the funding from DFG then does not materialise, in the worst case I cannot implement the study. If you want to cooperate with companies, it is the same problem: they want to have an approved protocol (*Tierversuchsantrag)*.
Due to staffing	R12	Q2	Especially in areas where clinicians do animal studies, they have a one year sabbatical or time window in which they have to do their research. They often do not manage that, or they manage to do the experiments but then they are back in the clinic and it doesn’t get published.
For scientific reasons	R04	Q3	The preparatory work–cell cultures, in vitro, whatever can be done without animals–is done in the time while you write the *Tierversuchsantrag*. And if the *Tierversuchsantrag* gets approved, but it becomes evident in the intervening time that this idea does not work, then you have the [approved] *Tierversuchsantrag* but it cannot be acted upon. Because the method behind it does not work.
**Experiment level tracking challenges**			
Maintain flexibility	R09	Q7	You don’t know what exactly you need at the point in time at which you write the *Tierversuchsantrag* and at times you design the *Tierversuchsantrag* too large or integrate flexibilities of which you know that they cannot be used at the end of the day.
Experiments not performed	R09	Q10	It can end up with me writing a group for day 2, day 3, day 4 into my *Tierversuchsantrag*. Because I don’t have a choice. (…) And then I discover that day 2 is excellent but day 3 is far too late because no cells can be found any more (…) That’s one clear case in which you have to say, it doesn’t make any scientific sense to conduct all approved experiments (…) So it can happen that I only used 30% of the [approved] animals.
Aenderungsantraege	R08	Q13	You would have to go through every *Aenderungsantrag* (…) That’s a lot of work, if it’s possible at all. And only then, I think, could you finally say how much underreporting there is.
**Animal level tracking challenges**			
Experiment terminated early	R13	Q14	You also always want to work in a way that is protective of animal welfare. (…) Just because I have approval for 100 animals does not mean that, if I notice after several experiments that it is futile, that I continue just because the protocol says so.
Reductions not documented	R04	Q12	Even after *Aenderungsanzeigen* only additional animals are recorded, when you applied for additional groups. But it doesn’t get recorded anywhere that conversely, you also no longer need other experimental groups.
**Literature matching challenges**			
Crossover of publications	R15	Q19	In a clinical trial you probably expect one trial per publication. . . Within animal research you could have ten different experiments in the same publication. And then trying to track down which methods actually correspond to which experiments and which result is really, really hard.
Saved for future publication	R03	Q21	If I had published this result in a low impact factor journal straight after my doctorate, just to also publish the negative result, as is desirable, I wouldn’t have had the opportunity two years later to include that as a control and thereby upgrade the [new] study so that I can publish it better [in a more highly ranked journal].
**CAUSES OF INCOMPLETE REPORTING**			
**Lack of incentives to report negative and null results**			
Hard to publish high impact	R02	Q26	If it’s a single, negative result, and you have another 20 papers to publish with positive results, then it is very likely which you will tackle first, because they promise a high impact and so on. (…) Do I aim at one big publication, to become visible, or do I–in inverted commas–“waste” time for the publication of negative results that possibly benefit other people in decades down the line? You can’t burden young ECRs with that. But at the end of the day, it’s them who have to generate the data and do the groundwork.
Fixation on p-values	R17	Q23	Because there is no asterix next to it, it doesn’t get published. But the observed effect can nonetheless be relevant.
Hard to publish replications	R05	Q28	In immunology and vaccine development (…) it’s a race against time. (…) But when I’ve found something similar or identical, it gets difficult to publish it again because it is no longer new. Or you can publish it again, but no longer in a highly ranked or equally ranked journal.
When can null results be published ‘well’	R02	Q29	Regarding the publication of negative data, it depends on how confrontational or spectacular a result is. If for example a certain mechanism was postulated for decades and now it is shown in an animal study that that is not the case, then you can surely publish such negative data very prominently.
**Pressures to deliver positive results**			
Selective reporting	R08	Q33	I think that’s the most frequent kind of fault, that animals are excluded. (…) I don’t stand behind every person using a pipette. But I believe that of course things like that happen. People are under pressure, they need a job, else they don’t have anything to do–there’s no need to deceive ourselves. And I believe that that can also lead to wrong [“*falschen*”] human trials.
**Perceptions that some data do not add value**			
Depends on context	R07	Q52	There are things that fall between the cracks where I would really absolutely say: It’s like that, I see no problem at all. And with other things: It gives me stomach aches [“*gibt mir Bauchschmerzen*”] that it doesn’t appear anywhere.
Data ruined	R16	Q35	There can be unforeseen events and those are the things where I then say, I don’t want them published as negative data. During the study the air conditioning failed… so there were unintentional influences that the best planning and competence could not prevent. (…) So, erroneous [“*fehrlerhafte*”] data are not data that advance science.
Dropout pre-measurement	R11	Q37	You only realise it afterwards, once you’re done the experiment, euthanised and autopsied them, looked at the bio-distribution, and then see: “What we applied didn’t reach its intended destination.” (…) So basically we have no result, because the [research] question had not been addressed.
Experiment terminated early after tiny pilot group	R13	Q39	If we have a *Tierversuchsantrag* with five groups, so and so many animals, at the end only three groups are pursued and the rest get dropped because you notice that this is not practicable or whatever. (…) Those are not real statistics, I say.
**Commercial pressures**			
Commercial influence	R14	Q41	If you work completely within contract research [outside a university] it’s different. There it’s: “I bought that and the study didn’t show that result, so it gets put on ice and possibly the study gets done again at a different site.” (…) Then the study is done three, four, five times with a different CRO until you get the result that you want. (…) And when it isn’t expedient, then it gets swept beneath the carpet rather than somehow being brought into connection with a product.
**Reputational concerns**			
May suggest study was flawed	R01	Q43	Was that a thought through hypothesis, or was that from the outset a hypothesis that cannot work at all?
Drop-out rates can reflect on competence	R15	Q45	People don’t want to say that their surgery is only effective 50 percent of the time (…) They want to give this impression that everything works all the time, but science is messy.
**Socio-political and regulatory pressures**			
Stigma and external pressure	R06	Q49	I think we need to get away from this, ‘I am being controlled or even punished’. Sometimes it’s scary. If you try to follow the rules, you actually are more afraid that someone could point the finger at you, because there is suddenly something to control. But if I don’t document anything, I run less danger.

Note: R = respondent number (interview partner); Q = number of quote as cited in the paper.

### Measurement challenges of incomplete reporting

Incomplete reporting of results can take place on three levels. Researchers can decide to not report the results of an entire *study*, or of discrete *experiments* nested within each study, or of individual *animals* used (see Table 1).

#### Study level tracking challenges

Respondents concurred that generating animal study cohorts from regulatory approvals (including amendments) and then searching the literature for related publications is an appropriate way to measure incomplete reporting at the study level, albeit with three caveats.

First, some commercial and non-commercial funders require approvals to be in place before reviewing funding applications, and some studies that receive regulatory approval subsequently fail to secure funding (Q1). Second, there is a substantial delay between filing a project evaluation (*Tierversuchsantrag*) with regional German authorities and receiving authorization; multiple respondents cited nine months as a typical time span, though this may vary by federal region (*Bundesland*) and individual study. Turnover of staff (Q2) or new scientific developments (Q3) during that waiting period may lead a study team to decide not to initiate an authorized project. Third, literature searches for animal study results face substantial challenges (see further below).

#### Experiment level tracking challenges

Respondents concurred that in the German context, application documents for project evaluations (*Tierversuchsantraege*) by themselves cannot be used to meaningfully and reliably measure incomplete publication *at the experiment level*.

Several respondents highlighted that the German project evaluation and authorization system requires all experiments to be specified in great detail many months before work on a study begins, while the exploratory nature of their research requires flexibility to modify the study design as work progresses and new insights emerge.

Respondents concurred that German researchers routinely seek to maintain this flexibility by crafting applications that incorporate a very wide range of possible experiments and a large number of animals, to cover possible future contingencies (Q4, Q5, Q6, Q7, Q8, Q50).

In vitro studies or early stage experiments often show an initially envisaged line of enquiry to be futile, or required compounds or materials cannot be secured. When this happens, the originally planned experiments are never performed (Q9, Q10).

Conversely, when a line of enquiry appears fruitful, new research questions may emerge. To be able to address those, researchers file requests to modify the original approvals (*Aenderungantraege*) by replacing predefined experiments with new ones, while keeping animal numbers constant. This way, an original application may be modified dozens of times (Q11). However, if discrete experiments are never performed, this is rarely reported back to the authorities (Q12).

Measuring incomplete reporting at the experiment level thus requires taking the original approval as the starting point, and then working sequentially through all subsequent *Aenderungantraege*. While time consuming (Q13), this can be used to establish an upper limit for the number of experiments that might have been performed, but not the precise number of experiments actually performed.

#### Animal level tracking challenges

Similarly, researchers may terminate experiments early, using fewer animals than planned (Q14, Q15), but such reductions are rarely reported back to the authorities (Q16, Q17). Therefore, applications for animal research (*Tierversuchsantraege*) in conjunction with subsequent modification requests (*Aenderungsantraege*) can be used to establish an upper limit for the number of animals that might have been used, but not the precise number of animals actually used.

#### Literature matching challenges

In human clinical trials, a single journal article typically describes the outcomes of a single trial. Tracing outcome publications for animal studies is far more challenging because a scientific research project can involve multiple applications for animal research (*Tierversuchsantraege*), and multiple experiments nested within several applications may later be recombined into a single scientific paper (Q18, Q19). In addition, some outcomes may only get published within doctoral theses or in other formats, or kept on file indefinitely until they can be ‘fitted’ into a future broader publication (Q20, Q21).

#### Causes of incomplete reporting

According to respondents, there are six drivers of incomplete reporting of results in animal research: a lack of incentives to report non-positive results, pressures to ‘deliver’ positive results, perceptions that some data do not add value, commercial pressures, reputational concerns, and socio-political and regulatory pressures.

#### Lack of incentives to report negative and null results

Respondents unanimously concurred that the lack of incentives for academic researchers to publish ‘negative’ or ‘null’ findings is a major driver of non-reporting at all levels: project, experiment and individual animal (Q22). High impact journals that are crucial to academics’ career progression and ability to attract future funding are commonly not interested (Q51) in publishing non-positive findings (commonly defined through p-values, Q23) or replications (Q28), regardless of methodological rigour or scientific merit (Q24). Some respondents mentioned that if a paper on a ‘positive’ project includes non-positive results for discrete experiments, editors or reviewers often remove these (Q25). Publication in lower impact journals is unattractive, mainly due to opportunity costs (Q26), but also because achieving tenure can hinge on a researcher having a high impact average across all publications (Q27).

However, some respondents noted that non-positive findings can be published high impact if they refute previous landmark findings in the field (Q29). While the evidence bar may be set higher in such cases, such papers can later attract many citations (Q31).

#### Pressures to deliver positive results

Career pressures to deliver clearly ‘positive’ results can drive some researchers to omit the data for some animals in journal articles (Q32). Respondents believed that such selective reporting is not uncommon (Q33, Q34).

#### Perceptions that some data do not add value

Furthermore, some respondents thought that reporting some data was unnecessary as it would not add any scientific value (Q52).

Examples cited included data ruined by laboratory accidents (Q35), pre-intervention and pre-measurement dropouts (Q36, Q37), unexplained failures due to unknown variables (Q38), and experiments terminated after only very few animals were used (Q39).

#### Commercial pressures

When studies are funded by commercial entities, funders may sometimes object to the publication of results because they are viewed as commercially confidential (Q42) or because they reflect negatively on the product being tested (Q41).

#### Reputational concerns

Some respondents also pointed out that an absence of ‘positive’ results could indicate that a study was badly conceived (Q43), and that having high drop-out rates pre-experiment could be interpreted as a lack of skills (such as surgical skills) by an individual or study team (Q44, Q45), potentially exposing researchers to criticism even when a study was well designed and implemented.

#### Socio-political and regulatory pressures

Such reputational concerns are compounded by legal and reputational pressures. Widespread public and political animosity towards animal research in Germany (Q46, Q47) and close monitoring by activist groups (Q48, Q49) can disincentivise the sharing of failures and ‘negative’ results.

## Discussion

### Measuring incomplete reporting

Generating meaningful and reliable data on the extent of incomplete publication of biomedical animal studies is challenging in the German context. At the *study level*, it requires verifying that approved studies were actually initiated post-approval and taking into account complex publication pathways. At the *experiment level* and *animal level*, it requires analysing approvals plus numerous modification requests (*Aenderungsantraege*). This time consuming methodology can establish an upper bound for the numbers of experiments performed and/or animals used, but will typically not capture post-approval reductions in the numbers of experiments of animals. To generate precise data on experiments performed and/or animals used, addition data, for example from laboratory notebooks, specific documentation of animal research facilities, or other source would be required.

### Causes of incomplete reporting

Respondents flagged six drivers of incomplete reporting of results in biomedical animal research. Four of these drivers–lack of incentives to report certain results, pressures to ‘deliver’ positive results, perceptions that some data do not add value, and commercial pressures–closely match drivers for incomplete reporting in other areas of research [18–22]. The fifth driver–reputational concerns–may play a far greater role in animal research than in human drug trials, possibly because investigators’ technical skills can affect animal survival rates more directly.

The sixth driver of incomplete reporting–socio-political and regulatory pressures–may be a specific feature of animal studies. The lack of social and political consensus in Germany on the desirability of conducting such research in the first place, combined with the vigilance of advocacy groups, generates an environment that discourages reporting the results of ‘failed experiments’ involving animals. In contrast, there is overwhelming social and political consensus that running well-designed clinical trials in humans is desirable, and a tacit understanding that clinical equipoise dictates that some participants in clinical trials may fail to experience benefits or even suffer harms.

The finding that reputational concerns are a strong driver of incomplete reporting in this field may merit further research. For example, surgeons’ skill and experience can affect the success rates for surgery [23, 24]. Future research could explore whether reputational concerns influence the reporting of clinical trials of surgical interventions.

### Publicly available information about individual animal experiments

Incomplete reporting of animal studies can be reliably quantified at the *study level* using approval documentation in Europe, as two previous studies have already done [13, 14]. Individual animal studies, however, mostly comprise several experiments with hundreds of animals. The reporting rate at the study level, therefore, is conceptually flawed as it only captures whether any results of any experiment with any animals were reported. For a more meaningful understanding of the extent of incomplete reporting in animal research, the measurement of results reporting at the level of individual experiments or individual animals is needed.

Efforts at precise quantification at the *experiment level* and *animal level*, however, would require additional data about what animal experiments are ultimately conducted. This information is currently not accessible for systematic evaluations. One potential source for this kind of information could be a local or national documentation about the characteristics of all animal experiments started and completed at university-based animal research facilities. The comprehensive preregistration of individual animal experiments could also facilitate measurements on publication rates as demonstrated for clinical research [25, 26]. Preregistration of animal studies, however, is still in its infancy [27, 28].

### Limitations

This study has two limitations. First, there may be additional factors contributing to incomplete reporting in animal research that were not identified by respondents. The interview guide asked the open-ended question of what the most common causes of incomplete reporting were, and we have reported all causes flagged by respondents. However, it is possible that additional, less common causes were not flagged during 18 hours of interviews. Second, it is unclear whether and to what extent its findings are generalizable beyond the specific context of animal research conducted within Germany only. EU countries conducting animal research under the directive 2010/63/EU might experience similar challenges for measuring publication rates if they apply a similar study application and approval system that integrate different animal experiments under the umbrella of one animal study.

### Concept for complete results reporting in animal research

Our research indicates that the concept of complete reporting in animal research remains contested and underdefined. While reporting guidelines such as ARRIVE (Animal research: reporting in vivo experiments) [29] reflect reporting quality further guidance is needed that specify what data out of all animal studies require reporting to guarantee an unbiased knowledge gain and what data do not merit reporting in this regard? Furthermore, what dissemination routes qualify as appropriate results reporting? Several journals explicitly invite the submission of “negative” or “undesired” results such as PLoS One or BMJ Open Science. Other journals certainly should follow this example to facilitate an unbiased results reporting. Beside peer-reviewed journal articles also preprints, data repositories, or summary results in publicly accessible registries/databases might become important formats for results dissemination. In clinical trials, for example, the reporting of summary results in trial registries has become a broadly accepted alternative to journal publication.

Future efforts to improve results reporting in animal studies should take into account socio-political pressures because in some contexts these can be significant factors discouraging the reporting of pre-experimental dropout rates and ‘null’ and ‘negative’ outcomes. More incentives and rewards, including career incentives, for complete results reporting in animal research might help to improve the status quo. Similar to the development of reporting guidelines [29, 30] or other guidelines from the Laboratory Animal Science Association (LASA) [31] the relevant stakeholder groups in animal research, including animal researchers, animal research facilities, funders, expert networks, and regulators should work together to develop guidance and best practice standards for comprehensive results reporting. Once such guidance is available the valid measurements of the extent and consequences of incomplete reporting in animal research should be facilitated by academic institutions and regulators.

## Supporting information

S1 File Anonymised list of interviewees. (DOCX)

S2 File Consent form. (DOCX)

S3 File Interview guide in German and English. (DOCX)

S4 File Quotes by interviewees. (DOCX)
