# Supplementary material for: Measurement challenges and causes of incomplete results reporting of biomedical animal studies: Results from an interview study
Source: PLoS One. 2022 Aug 12;17(8):e0271976. doi: 10.1371/journal.pone.0271976 (PMC9374215; doi:10.1371/journal.pone.0271976)
Supplement: S1 File — (DOCX) [file pone.0271976.s001.docx]

Supplement 1, List of interviewees

Bruckner T, Wieschowski S, Heider M, Deutsch S, Drude N, Tölch U, Bleich A, Tolba R, Strech S (preprint) Measurement challenges and causes of incomplete results reporting of biomedical animal studies: Results from an interview study

| **Code** | **Type** | **Country** |
| --- | --- | --- |
| R01 | Animal researcher | Germany |
| R02 | Animal researcher | Germany |
| R03 | Animal researcher | Germany |
| R04 | Animal researcher | Germany |
| R05 | Animal researcher | Germany |
| R06 | Animal researcher | Germany |
| R07 | Animal researcher | Germany |
| R08 | Animal researcher | Germany |
| R09 | Animal researcher | Germany |
| R10 | Animal research methodology expert | UK |
| R11 | Industry group representative | Germany |
| R12 | Animal welfare officer and researcher | Germany |
| R13 | Animal researcher | Germany |
| R14 | Animal welfare officer and researcher | Germany |
| R15 | Animal research methodology expert | UK |
| R16 | Animal researcher | Germany |
| R17 | Animal researcher and statistician | Germany |
| R18 | Journal editor | Germany |
