# Supplementary material for: Measurement challenges and causes of incomplete results reporting of biomedical animal studies: Results from an interview study
Source: PLoS One. 2022 Aug 12;17(8):e0271976. doi: 10.1371/journal.pone.0271976 (PMC9374215; doi:10.1371/journal.pone.0271976)
Supplement: S2 File — (DOCX) [file pone.0271976.s002.docx]

Supplemental2: Consent form

**EMBARC – Extent, Predictors, and Management of Publication Bias in Animal Research**

*Interviewstudie zum Thema „Ergebnisveröffentlichung von Tierstudien“*

**Studieninformation**

**Studienleitung**

Prof. Dr. med. Dr. phil. Daniel Strech

QUEST Center (Quality, Ethics, Open Science, Translation)

Berlin Institute of Health (BIH)

Translationsforschungsbereich der Charité – Universitätsmedizin Berlin

Anna-Louisa-Karsch-Str. 2, 10178 Berlin

Tel: +49 30 450 543-068/676

Email: [daniel.strech@charite.de](mailto:daniel.strech@charite.de)

**Förderung**

BMBF-Förderlinie „Alternativmethoden zum Tierversuch“

**Hintergrund unserer Studie**

In Phase 1 von EMBARC wurde die Publikations-Rate in Tierversuchsstudien quantitativ erforscht und dokumentiert. Für eine deutsche Kohorte von 210 abgeschlossenen Tierversuchsanträgen wurde in 70% der Fälle durch Datenbankrecherche und Anschreiben der Studienverantwortlichen mindestens eine Ergebnispublikation gefunden. Allerdings wird in den Ergebnispublikationen oft eine Anzahl von Tieren genannt, die geringer ist als die Anzahl der genehmigten Tiere.

In Phase 2 von EMBARC wollen wir ein besseres Verständnis der Thematik erlangen. Vor allem wollen wir erkunden, was unter einer „vollständigen Ergebnisveröffentlichung“ in der Tierforschung sinnvollerweise verstanden werden kann. Anhand unserer Stichprobe von 210 Tierversuchsanträgen haben wir in Phase 1 von EMBARC die Publikationsrate auf drei verschiedene Arten berechnet

1. Antrags-Level: Publikationen pro Tierversuchsantrag (ca. 70%) ([Publikation hier](https://www.ncbi.nlm.nih.gov/pmc/articles/PMC6879110/)):
2. Experiment-Level: Anteil der Experimente aus dem Antrag in Publikationen ca. 40% (vorläufige Daten)
3. Tier-Level: Anteil der Tiere aus dem Antrag in Publikationen ca. 20% (vorläufige Daten)

Dieses Ergebnis wirft die Frage auf, wie die „vollständige Ergebnisveröffentlichung“ in der Tierforschung sinnvoll konzipiert werden könnte. Zudem wollen wir mögliche Strategien für eine noch vollständigere Ergebnisveröffentlichung untersuchen.

**Methodik**

Zu diesem Ziel werden wir semi-strukturierte Interviews mit ca. 20 Expertinnen und Experten zum Thema „Ergebnisveröffentlichung von Tierstudien“ durchführen. Teilnehmenden wird eine Aufwandsentschädigung in Höhe von 150 Euro angeboten. Die Studie dient wissenschaflichen Zwecken.

Die Interviews werden mit MS Teams aufgezeichnet, anonymisiert gespeichert und sodann von einem externen Dienstleister für Transkription in Schriftform gebracht. Für die weitere wissenschaftliche Auswertung der Interviewtexte werden alle Angaben, die zu einer Identifizierung der Person oder ihrer Institution führen könnten, verändert oder aus dem Text entfernt. Die Interviewdaten werden separat von Ihrem Namen und ihrer E-Mail Adresse gespeichert.

In wissenschaftlichen Veröffentlichungen werden Interviews nur in Ausschnitten zitiert, um gegenüber Dritten sicherzustellen, dass der entstehende Gesamtzusammenhang von Ereignissen nicht zu einer Identifizierung der Person oder ihrer Institution führen kann.

Bei Fragen zum wissenschaftlichen Hintergrund der Studie kontaktieren Sie bitte:

Dr. Susanne Wieschowski [wieschowski.susanne@charite.de](mailto:wieschowski.susanne@charite.de)

**Hinweis zum Datenschutz**

Die Interviewtranskripte werden in pseudonymisierter Form auf passwortgeschützen Servern der Charité – Universitätsmedizin Berlin gespeichert und verarbeitet werden, in Konformität mit der EU-Datenschutzgrundverordnung (DS-GVO) und anderer Gesetzgebung. Nur Mitglieder des Studienteams werden Zugriff auf diese Daten haben.

Nach Abschluss der Studie werden die Daten entsprechend den Prinzipien der guten wissenschaftlichen Praxis für zehn Jahre gespeichert; anschließend werden sie gelöscht.

Datenschutzbeauftragter: Fragen über die Speicherung und Bearbeitung Ihrer Daten, oder über Ihre Rechte auf Datenschutz, können Sie jederzeit an den Datenschutzbeauftragten der Charité richten: Datenschutz der Charité – Universitätsmedizin Berlin, Charitéplatz 1, 10117 Berlin, Tel: +49 30 450 580 016, Email: [datenschutz@charite.de](mailto:datenschutz@charite.de)

Einspruchsrecht mit dem Beauftragten für Datenschutz und Informationsfreiheit: Sie haben das Recht, bei der zuständigen Behörde Einspruch einzulegen, falls Sie den Eindruck haben, dass die Verarbeitung Ihrer Daten nicht gesetzeskonform ist: Berliner Beauftragte für Datenschutz und Informationsfreiheit (Friedrichstr. 219, 10969 Berlin, Tel: +49 30 13889-0, E-Mail: [mailbox@datenschutz-berlin.de](mailto:mailbox@datenschutz-berlin.de))

**Einwilligungserklärung**

**Ich erkläre mich zur Teilnahme an einem semi-strukturierten Interview im Rahmen der EMBARC Phase 2 Studie bereit.** Mir ist bekannt, dass meine Teilnahme freiwillig ist, dass mir bei Nichtteilnahme kein Nachteil entsteht, und das ich meine Teilnahme jederzeit ohne Angabe von Gründen abbrechen oder widerrufen kann.

Ich habe die obigen Informationen gelesen und verstanden und erkläre mich mit der Teilnahme an diesem Projekt einverstanden.

__________________ ____________ ____________________________________________

NAME DATUM UNTERSCHRIFT

**Consent form – English translation**

**Study information**

**Study Management**

Prof. Dr. med. Dr. phil. Daniel Strech

QUEST Center (Quality, Ethics, Open Science, Translation)

Berlin Institute of Health (BIH)

Translational Research Unit of the Charité - Universitätsmedizin Berlin

Anna-Louisa-Karsch-Str. 2, 10178 Berlin

Phone: +49 30 450 543-068/676

Email: daniel.strech@charite.de

**Funding**

BMBF funding line "Alternative methods to animal experiments

**Background of our study**

Phase 1 of EMBARC quantitatively explored and documented the publication rate in animal experimentation studies. For a German cohort of 210 completed animal experiment applications, at least one results publication was found in 70% of the cases through database searches and letters to study investigators. However, the results publications often mention a number of animals that is lower than the number of approved animals.

In Phase 2 of EMBARC, we want to gain a better understanding of the issue. In particular, we want to explore what can be meaningfully understood by a "full results publication" in animal research. Using our sample of 210 animal research applications, we calculated publication rates in three different ways in Phase 1 of EMBARC.

1. application level: publications per animal experiment application (approximately 70%) ([publication here](https://www.ncbi.nlm.nih.gov/pmc/articles/PMC6879110/)):

2. experiment level: proportion of experiments from the application in publications approx. 40% (preliminary data).

3. animal level: proportion of animals from the application in publications approx. 20% (preliminary data).

This result raises the question of how "full results publication" in animal research could be meaningfully conceptualized. In addition, we want to explore possible strategies for even more complete results publication.

**Methodology**

To this end, we will conduct semi-structured interviews with approximately 20 experts on the topic of "results publication in animal studies". Participants will be offered an expense allowance of 150 Euros. The study serves scientific purposes.

The interviews will be recorded with MS Teams, stored anonymously and then transcribed into written form by an external transcription service provider. For further scientific analysis of the interview texts, all information that could lead to an identification of the person or their institution will be changed or removed from the text. The interview data are stored separately from your name and e-mail address.

In scientific publications, interviews are quoted only in excerpts to ensure vis-à-vis third parties that the emerging overall context of events cannot lead to an identification of the person or their institution.

For questions regarding the scientific background of the study, please contact:

Dr. Susanne Wieschowski wieschowski.susanne@charite.de

**Note on data protection**

Interview transcripts will be stored and processed in pseudonymized form on password-protected servers at Charité - Universitätsmedizin Berlin, in compliance with the EU General Data Protection Regulation (GDPR) and other legislation. Only members of the study team will have access to these data.

After completion of the study, the data will be stored in accordance with the principles of good scientific practice for ten years, after which they will be deleted.

Data Protection Officer: Questions about the storage and processing of your data, or about your rights to data protection, can be directed to the Charité Data Protection Officer at any time: Data Protection of Charité - Universitätsmedizin Berlin, Charitéplatz 1, 10117 Berlin, Tel: +49 30 450 580 016, Email: datenschutz@charite.de

Right of appeal with the Commissioner for Data Protection and Freedom of Information: You have the right to appeal to the competent authority if you have the impression that the processing of your data does not comply with the law: Berlin Commissioner for Data Protection and Freedom of Information (Friedrichstr. 219, 10969 Berlin, Tel: +49 30 13889-0, Email: mailbox@datenschutz-berlin.de)

**Declaration of consent**

I agree to participate in a semi-structured interview as part of the EMBARC Phase 2 study. I understand that my participation is voluntary, that I will not be disadvantaged if I do not participate, and that I may terminate or withdraw my participation at any time without giving reasons.

I have read and understood the above information and agree to participate in this project.

__________________ ____________ ____________________________________________

NAME DATE SIGNATURE

Translated with www.DeepL.com/Translator (free version)
