# Supplementary material for: Measurement challenges and causes of incomplete results reporting of biomedical animal studies: Results from an interview study
Source: PLoS One. 2022 Aug 12;17(8):e0271976. doi: 10.1371/journal.pone.0271976 (PMC9374215; doi:10.1371/journal.pone.0271976)
Supplement: S3 File — (DOCX) [file pone.0271976.s003.docx]

Supplemental3: EMBARC Interview guide

Bruckner T, Wieschowski S, Heider M, Deutsch S, Drude N, Tölch U, Bleich A, Tolba R, Strech S (preprint) Measurement challenges and causes of incomplete results reporting of biomedical animal studies: Results from an interview study

**Einführung**

- Dank für Teilnahme
- Stelle Dich vor
- Inhalte des Interviews werden nur in pseudonymisierter Form veröffentlicht werden
- Zustimmung zur Audioaufnahme des Gesprächs? 🡪 Einwilligungsformular sollte vorliegen

***[Recorder einschalten]***

- Das Gespräch wird ab jetzt mit Ihrer Zustimmung aufgenommen
- Zweite Phase des EMBARC-Projektes geleitet von Daniel Strech
- Erste Phase: 210 Tierversuchsanträge für Experimentalstudien, etwa 2/3 publiziert

[Ggf. auf Nachfrage erläutern, dass es sich um Studien zu folgenden Zwecken laut §7a Abs 1. TierSchG): 1.Grundlagenforschung, 2. Vorbeugen, Erkennen oder Behandeln von Krankheiten, Leiden, Körperschäden oder körperlichen Beschwerden bei Menschen oder Tieren

Oder kurz: Grundlagenforschung und präklinische Forschung]

- - Aber: Unser Ansatz hatte vielleicht wichtige Aspekte der Tierforschung in der Praxis nicht berücksichtigt
- Ziel jetzt: Besser die Komplexität des Themas verstehen
  - Circa 20 semi-strukturierte Experten-Interviews
- Bitte ausschweifend antworten
  - Gerne mit Beispielen
  - Gerne auch wichtige Themen ansprechen, die von uns eventuell gar nicht berücksichtigt wurden
  - Falls Sie eine Frage nicht beantworten wollen ist das OK

**Thema 1. Definition der Publikationsrate**

Publikationsrate wie berechnen?

In der ersten EMBARC Phase haben wir die Publikationsrate auf drei verschiedene Arten berechnet:

***Antrags-Level***: Publikationen pro Tierversuchsantrag ca. 70%

***Experiment-Level***: Zahl der Experimente im Antrag in Publikationen ca. 40%

***Tier-Level***: Zahl der Tiere im Antrag in Publikationen ca. 20%

- Welche dieser drei Berechnungen halten Sie für die relevantesten für eine aussagekräftige Kalkulation der Publikationsrate , und warum?
- Welchen Wert sehen Sie in den zwei anderen Berechnungsarten?
  - In welchem Kontext?

Relevanz der Publikationsrate?

- Wenn z.B. 100 Tiere verwendet werden, aber die Ergebnisse von nur 20 Tieren danach veröffentlicht werden, was sind die häufigsten Gründe dafür?
- Wenn z.B. 100 Tiere verwendet werden, aber die Ergebnisse von nur 20 Tieren danach veröffentlicht werden, unter welchen Umständen könnte dies potenziell wissenschaftlich oder ethisch bedenklich sein?
  - Gibt es Kontexte, in denen dies überhaupt nicht bedenklich ist?
- Sind Definitionen von „vollständigen Ergebnisveröffentlichung“ aus klinischen Versuchen mit Menschen in die Tierforschung übertragbar? [Falls notwendig Helsinki erklären]
- Wie könnte man dieses Konzept an die Tierforschung anpassen?

**2. Mögliche Ansatzpunkte und Lösungsansätze**

Ansatzpunkte?

- Welche Ansatzpunkte sehen Sie, die Publikationsrate in der Tierforschung in der Praxis zu erhöhen?
- Welche potentiellen Ansätze wären konstruktiv, und welche sollten vermieden werden?
  - Falls keine Beispiele genannt werden, konkret diese Ideen ansprechen:
    - Möglichkeit der Veröffentlichung von kurzen Zusammenfassungen der Ergebnisse („Summary Results“) in Registern für Tierforschung (ähnlich wie bei klinischen Studien)
    - Übernahme der Publikationskosten für Open Access Journale durch die Hochschulen oder Drittmittelgeber
    - Spezielle Leistungsorientierte Mittel (LOM) für die Veröffentlichung von tierexperimentellen Ergebnissen, die die ursprüngliche Hypothese nicht unterstützen oder von explorativen Studien, die keine Hypothese getestet haben (inkl. Publikationen auf Preprint-Servern oder Repositorien etc.)
    - Mehr Informationen zu Fachzeitschriften, die auch nicht-signifikante Ergebnisse aus Tierexperimenten berichten (z.B. PLoS One)

Lösungsansätze?

- Könnte eine „vollständige Ergebnisveröffentlichung” mit bestehenden Prozessen, Strukturen und Ressourcen erreicht werden?
  - Wenn nein: Welche zusätzlichen Prozesse, Strukturen und Ressourcen wären notwendig?
- Welche Akteure sollten oder könnten dabei eine Rolle spielen?
  - Welche Akteure sind nicht relevant, oder sollten nicht einbezogen werden?

**Schlussfragen**

- Gibt es Anliegen oder Überlegungen Ihrerseits, die wir bisher nicht angesprochen haben?
- Mit welchen weiteren Experten sollten wir sprechen, um die Thematik besser zu verstehen?

**Interview guide – English translation**

**Introduction**

- Thanks for participation
- Introduce yourself
- Contents of the interview will be published only in pseudonymous form
- Consent to audio recording of the interview? Consent form should be available

***[Switch on recorder]***

- The interview will be recorded from now on with your consent
- Second phase of the EMBARC project led by Daniel Strech
- First phase: 210 animal applications for experimental studies, about 2/3 published

[If necessary, explain on request that these are studies for the following purposes according to §7a Abs 1. TierSchG): 1. basic research, 2. prevention, detection or treatment of diseases, suffering, bodily harm or physical complaints in humans or animals.

Or in short: basic research and preclinical research]

- But: our approach had perhaps not considered important aspects of animal research in practice
- Goal now: Better understand the complexity of the topic
  - Approximately 20 semi-structured expert interviews.
- Please answer in detail
  - Gladly with examples
  - Feel free to address important issues that we may not have considered at all
  - If you do not want to answer a question that is OK

**Topic 1. definition of publication rate**

Publication rate - how to calculate?

- In the first EMBARC phase we calculated the publication rate in three different ways:

1. **application-level**: publications per animal experiment application about 70%.

2**. experiment level**: number of experiments in the application in publications approx. 40%.

3. **animal level**: number of animals in the application in publications approx. 20%.

- Which of these three calculations do you consider the most relevant for a meaningful calculation of the publication rate , and why?
- What value do you see in the other two types of calculations?
  - In what context?

Relevance of publication rate?

- For example, if 100 animals are used but the results of only 20 animals are published afterwards, what are the most common reasons for this?
- For example, if 100 animals are used but the results of only 20 animals are published afterwards, under what circumstances might this be potentially scientifically or ethically questionable?
  - Are there contexts in which this is not a concern at all?
- Are definitions of "full results publication" from human clinical trials transferable to animal research? [Explain Helsinki if necessary.]
  - How could this concept be adapted to animal research?

**2. possible starting points and solutions**

Starting points?

- What starting points do you see to increase the publication rate in animal research in practice?
- Which potential approaches would be constructive, and which should be avoided?
- If no examples are given, specifically address these ideas:
  - Possibility of publishing short summaries of results ("Summary Results") in registries for animal research (similar to clinical trials).
  - Payment of publication costs for open access journals by universities or third-party funders
  - Dedicated performance-based funding (LOM) for publication of animal experimental results that do not support the original hypothesis or exploratory studies that did not test a hypothesis (including publications on preprint servers or repositories, etc.)
  - More information on journals that also report non-significant results from animal experiments (e.g. PLoS One).

Possible solutions?

- Could "full results publication" be achieved with existing processes, structures, and resources?
  - If no: What additional processes, structures, and resources would be needed?
- Which actors should or could play a role in this?
  - Which actors are not relevant, or should not be involved?

**Concluding Questions**

- Are there concerns or considerations on your part that we have not yet addressed?
- What other experts should we talk to in order to better understand the issue?

Translated with www.DeepL.com/Translator (free version)
