# Supplementary material for: Measurement challenges and causes of incomplete results reporting of biomedical animal studies: Results from an interview study
Source: PLoS One. 2022 Aug 12;17(8):e0271976. doi: 10.1371/journal.pone.0271976 (PMC9374215; doi:10.1371/journal.pone.0271976)
Supplement: S4 File — (DOCX) [file pone.0271976.s004.docx]

Supplemental 4: EMBARC interview quotes

Bruckner T, Wieschowski S, Heider M, Deutsch S, Drude N, Tölch U, Bleich A, Tolba R, Strech S (preprint) Measurement challenges and causes of incomplete results reporting of biomedical animal studies: Results from an interview study

The quotes below are listed following the structure of the paper:

MEASUREMENT OF INCOMPLETE REPORTING

- Study level tracking challenges
- Experiment level tracking challenges
- Animal level tracking challenges
- Literature matching challenges

CAUSES OF INCOMPLETE REPORTING

- Lack of incentives to report negative and null results
- Pressures to deliver positive results
- Perceptions that some data do not add value
- Commercial pressures
- Reputational concerns
- Socio-political and regulatory pressures

***Note:***

***R = respondent number (interview partner)***

***Q = number of quote cited in text of the paper***

|  | | | **MEASUREMENT OF INCOMPLETE REPORTING** | |  |
| --- | --- | --- | --- | --- | --- |
| **Study level tracking challenges** | | | | | **Translation (part or whole)** |
| Funding not secured | R07 | Q1 | | Es ist bei uns viel an die Forschungsgelder gekoppelt. Bei der DFG zum Beispiel ist es so: Man muss einen Tierversuchsantrag mit vorgeben, der muss genehmigt sein. Wenn die Gelder jetzt aber bei der DFG doch nicht kommen, kann ich diesen Tierversuchsantrag im schlimmsten Falle gar nicht umsetzen. (…) Wenn man mit Firmen kooperieren möchte und so weiter, ist ja auch das Problem: Die wollen einen fertigen Tierversuchsantrag haben. | For example with [German public funder] DFG you have to submit a [pre-] approved protocol (Tierversuchsantrag). When the funding from DFG then does not materialise, in the worst case I cannot implement the study. If you want to cooperate with companies, it is the same problem: they want to have an approved protocol (Tierversuchsantrag). |
| Due to staffing | R12 | Q2 | | Wenn mir auch noch in der Arbeitsgruppe jemand ausfällt [und] (…) jemand anderes kann es nicht ausreichend gut. Vor allem in den Bereichen, in denen Kliniker Tierversuche machen, die haben ihr Sabbatical für ein Jahr oder ein forschungsfreies Zeitfenster, in dem müssen sie dann diese Versuche machen. Das schaffen sie häufig nicht oder sie schaffen es, die Versuche zu machen, sind dann aber wieder in der Klinik und dann wird das nicht publiziert. | Especially in areas where clinicians do animal studies, they have a one year sabbatical or time window in which they have to do their research. They often do not manage that, or they manage to do the experiments but then they are back in the clinic and it doesn’t get published. |
| For scientific reasons | R04 | Q3 | | Die Vorarbeiten – Zellkultur oder was auch immer , alles in vitro, was ohne Tier geht, macht man ja in der Zeit, wo man den Antrag schreibt. Und wenn dann der Antrag durch ist, aber dann sich rausstellt in dieser Zwischenzeit, dass diese Idee nicht funktioniert, dann ist der Antrag da, kann aber nicht bearbeitet werden. Weil die Methode dahinter nicht funktioniert. | The preparatory work – call cultures, in vitro, whatever can be done without animals – is done in the time while you write the *Tierversuchsantrag*. And if the *Tierversuchsantrag* gets approved, but it becomes evident in the intervening time that this idea does not work, then you have the [approved] *Tierversuchsantrag* but it cannot be acted upon. Because the method behind it does not work. |
| **Experiment level tracking challenges** | | | | |  |
| Maintain flexibility | R08 | Q4 | | Tierversuchsanträge von meiner Gruppe haben klassischerweise zwischen 4.000 und 5.000 Tiere. Die haben klassischerweise Teil A bis N, oder L, oder M. Also die sind ganz kleinteilig. Wir haben klassischerweise auch Gruppen drin... eingepflegt, um sie später austauschen zu können. | *Tierversuchsantraege* from my group typically have between 4,000 and 5,000 animals. They typically have parts [sections] A to N. So they are very granular. We typically also have groups in there … to enable them to later be substituted. |
| Maintain flexibility | R08 | Q5 | | Also ja, wir beantragen selbstverständlich nicht, was wir machen wollen, sondern wir stellen uns vor: Was könnten wir denn machen müssen, für dieses Projekt? Und so wird es beantragt. Das sind Riesenanträge mit vielen tausend Tieren. Und am Ende würde ich denken, dass wir im Schnitt nur ein Viertel der Tiere verbrauchen. (…) Die gehen immer davon aus, dass Sie wissen, was Sie tun. Aber genau das Gegenteil ist der Fall, – wir wissen per definitionem nicht, was wir tun. Sonst wären wir ja keine Wissenschaftler. | We obviously [“*selbstverstaendlich*”] do not apply for what we want to do, but we imagine: What might we have to do, for this project? Und that’s how it gets submitted. Those are huge *Tierversuchsantraege* with thousands of animals. And in the end, I think, we will only use a quarter of the animals. (…) They always assume that you know what you are doing. But exactly the opposite is the case – by definition we do not know what we are doing. Otherwise we wouldn’t be scientists. |
| Maintain flexibility | R06 | Q50 | | Man weiß, man wird Änderungsanträge schreiben müssen (…) dass man eine gewisse Flexibilität hat. Das hat sich so entwickelt, weil man gezwungen wird, da das System so unflexibel ist. | You know that you will have to write *Aenderungsanzeigen* (…) to have a certain flexibility. That has developed because you are forced [to do it], because the system is so inflexible. |
| Maintain flexibility | R07 | Q6 | | das haben wir alles beantragt, weil man ja auch nur eine bestimmte Anzahl an Tieren nachträglich nachtragen darf, noch mal, wenn es Änderungsanzeigen gibt | We applied for all that because you are only allowed to subsequently apply for a certain number of additional animals [in the framework of *Aenderungsanzeigen*]. |
| Maintain flexibility | R09 | Q7 | | Also ein Problem ist, dass man nicht genau weiß, was man braucht zu dem Zeitpunkt, wo man den Antrag schreibt und teilweise Anträge zu groß gestaltet oder Flexibilitäten hineindenkt, von denen man weiß, dass man die am Ende des Tages aus verschiedenen Gründen niemals benutzen kann. Es ist ja auch völlig klar, wenn ich einen Tierversuchsantrag schreibe, und da werden 1000 Tiere genehmigt, ist es doch meistens völlig unrealistisch. Also wenn ich mir überlege, was ist der Zeit- und Kostenaufwand, diese Tiere zu untersuchen, dann ist es völlig evident, dass das häufig gar nicht möglich sein wird. | You don’t know what exactly you need at the point in time at which you write the *Tierversuchsantrag* and at times you design the *Tierversuchsantrag* too large or integrate flexibilities of which you know that they cannot be used at the end of the day. It is absolutely clear that if I write a *Tierversuchsantrag* und 1,000 animals are approved, that that is usually completely unrealistic. If I consider what the time and cost of investigating those animals would be, then it is totally evident that that will often be impossible. |
| Maintain flexibility | R11 | Q8 | | Es gibt durchaus Anreize in dem ganzen System, dass man Versuche beantragt und genehmigt bekommt, auch sozusagen prospektiv, in die Zukunft gedacht, verschiedene Möglichkeiten abdeckt, die man verfolgen möchte, die dann aber zum Teil eben nicht verfolgt werden können aus Kapazitätsgründen oder weil sich der Forschungsfokus mit gewissen Experimenten oder Ergebnissen immer wieder verschiebt und anpasst. Wenn die Anträge auf drei oder sogar auf fünf Jahre in die Zukunft gestellt sind, dass sich dann ja auch vielleicht die Personalzusammensetzung ändert, dass das Projekt sich doch in eine andere Richtung entwickelt; dass dann bestimmte Versuche nicht durchgeführt werden. | There really are incentives in the whole system to submit and receive approval for *Tierversuchsantraege*, including so to say prospectively, thinking into the future, [that] cover different possibilities that one may want to pursue, but that then partially cannot be followed up on due to capacity constraints or because certain experiments or results repeatedly shift and adjust the focus of research. When *Tierversuchsantraege* are aimed at three or even five years into the future, then staffing might also have changed, and that the project develops into a different direction and certain experiments are not performed. |
| Experiments not performed | R03 | Q9 | | Manchmal haben wir Teilprojekte auch gar nicht erst in Angriff genommen, weil sich dann halt nach dem ersten herausgestellt hatte „Das ist eine Sackgasse oder so geht es nicht weiter“, | Sometimes we haven’t started project components because we discovered after the first [experiment(s)] that this is a dead end and will not take us any further. |
| Experiments not performed | R09 | Q10 | | dann kann es dadurch so enden, dass ich tatsächlich in meinem Tierversuchsantrag dann eine Gruppe Tag 2, Tag 3, Tag 4 reinschreibe. Weil ich kann ja nicht anders. (…) Ich habe dann die Genehmigung und fange an, die Versuche durchzuführen. Und dann stelle ich relativ schnell fest, dass vielleicht Tag 2 super ist, aber Tag 3 ist viel zu spät, ist sind nicht mehr genügend Zellen zu finden. (…) also das ist ein ganz klarer Fall, wo man sagen muss, das macht wissenschaftlich gar keinen Sinn, alle diese Versuche, die da beantragt wurden, tatsächlich so durchzuführen, wie sie beantragt waren. (…) kann das schnell sein, dass ich nur 30 Prozent der Tiere überhaupt benutzt habe. | It can end up with me writing a group for day 2, day 3, day 4 into my *Tierversuchsantrag.* Because I don’t have a choice. (…) And then I discover that day 2 is excellent but day 3 is far too late because no cells can be found any more (…) That’s one clear case in which you have to say, it doesn’t make any scientific sense to conduct all approved experiments (…) So it can happen that I only used 30% of the [approved] animals. |
| Aenderungsantraege | R08 | Q11 | | Wir arbeiten mit Tierversuchsänderungen, im Wesentlichen. Das geht so weit, dass wir manchmal pro Woche mehrere Tierversuchsänderungen rausschicken… im Allgemeinen zwischen einer Woche und vier Wochen kriegen wir da eine Antwort, selten eine Rückfrage. Und damit können wir arbeiten. | We mainly work with *Aenderungsantraege*. It goes so far that we sometimes send out several *Aenderungsantraege* per week… generally we get a reply within 1-4 weeks, rarely a request for further information. And we can work with that. |
| Aenderungsantraege | R08 | Q13 | | dann müssten Sie jeden Änderungsantrag durchgehen, um sicher zu sein, welche Versuche genau gemacht wurden. (…) Das ist viel Arbeit, wenn es überhaupt möglich ist. Und erst dann könnte man, glaube ich, schlüssig sagen, wie viel Underreporting da ist. | You would have to go through every *Aenderungsantrag* (…) That’s a lot of work, if it’s possible at all. And only then, I think, could you finally say how much underreporting there is. |
| **Animal level tracking challenges** | | | | |  |
| Experiment terminated early | R13 | Q14 | | man will ja auch immer ein bisschen tierschonend arbeiten. Nur, weil ich die 100 Tiere beantragt habe, heißt das nicht, dass ich, wenn ich jetzt nach so und so viel Versuchen schon merke, dass das nichts wird, dass ich dann weitermache, nur weil es das Protokoll vorgibt. Ich glaube, das ist nicht die richtige Herangehensweise und so soll es ja auch dann nicht sein, sondern wenn man merkt, man geht in die falsche Richtung, macht man es nicht weiter. | You also always want to work in a way that is protective of animal welfare. (…) Just because I have approval for 100 animals does not mean that, if I notice after several experiments that it is futile, that I continue just because the protocol says so. (…) I don’t think that’s the right approach and it shouldn’t be that way. Instead, when you notice it’s going in the wrong direction, you do not continue. |
| Experiment terminated early | R14 | Q15 | | Der Tierschutzbeauftragte ist natürlich schon so ein bisschen, wenn es schlecht läuft derjenige, der das Stoppschild hoch setzt und sagt „Nee, jetzt brechen wir hier ab“. | The animal protection officer [within the institution] is of course, somewhat, the person who holds up a stop sign and says, ‘no, this is where we stop’. |
| Reductions not documented | R04 | Q12 | | Also es wird auch nach Änderungsanzeigen immer nur ein Mehr an Tieren erfasst, wenn man zusätzliche Gruppen beantragt hat. Es wird aber nirgendwo erfasst, dass man dafür ja anderer Stelle auch Versuchsgruppen nicht mehr braucht. | Even after *Aenderungsanzeigen* only additional animals are recorded, when you applied for additional groups. But it doesn’t get recorded anywhere that conversely, you also no longer need other experimental groups. |
| Reductions not documented | R11 | Q16 | | Es gibt ja überhaupt keine Anreize, einen Änderungsantrag zu schreiben, wenn weniger Tiere eingesetzt werden. Man sagt ja "Never say never" und man weiß ja nicht, vielleicht wird [das] Experiment doch noch mal irgendwann relevant. Und warum sollte man sich absichtlich der Möglichkeit berauben, wenn dafür gar keine Notwendigkeit besteht? (…) für die Behörde ist es letztlich irrelevant beziehungsweise wäre ein erheblicher Mehraufwand, das nachzuhalten, in irgendeiner Form. | There are absolutely no incentives to write an *Aenderungsantrag* when less animals are used. One says ‘never say never,’ and you don’t know, maybe the experiment will sometime again become relevant. And why should you intentionally rob yourself of the ability if there is no necessity to? (…) For the regulatory body it is in final analysis irrelevant, or considerable additional effort, to record that in any form. |
| Reductions not documented | R12 | Q17 | | Häufig machen sich dann die Arbeitsgruppen nicht die Mühe und geben den Versuch wieder zurück, dass sie sagen: „Das hat nicht funktioniert. Liebe Genehmigungsbehörde, wir verzichten auf die Durchführung der Versuche.“ Den Aufwand machen sie sich einfach nicht. | Often the working groups do not make the effort to give the experiment back, to say: ‘That didn’t work. Dear regulator, we dispense with implementing the experiments.’ They simply don’t make that effort. |
| **Literature matching challenges** | | | | |  |
| Crossover of publications | R09 | Q18 | | Also wir haben wahrscheinlich im Institut, ich weiß nicht, zu einem Zeitpunkt, keine Ahnung, 10, 20 laufende Tierversuchsanträge. Und in dem Augenblick überschneiden sich die Dinge natürlich auch. Ein Projekt ist nicht ein wissenschaftliches Projekt, ist nicht eins zu eins in einem einzigen dazugehörigen Tierversuchsantrag abgebildet. Vielleicht gibt es drei oder vier Tierversuchsanträge, die alle anteilig zu einem wissenschaftlichen Projekt gehören. | We probably have around 10, 20 running *Tierversuchsantraege* at my institute at any given point in time. And at that of course things overlap. A scientific project is not represented one-to-one in a single corresponding *Tierversuchsantrag*. Maybe there are three or four *Tierversuchsantraege* that all form part of one scientific project. |
| Crossover of publications | R15 | Q19 | | in a clinical trial you probably expect one clinical, like one trial per publication because you know you are talking about big trials. Within animal research you could have ten different experiments in the same publication. And then trying to track down kind of like you know which methods actually correspond to which experiments and which result. I mean that’s it’s really, really hard. (…) even tracking down what papers result from which grant is quite, you know, is actually not that straight forward | In a clinical trial you probably expect one trial per publication... Within animal research you could have ten different experiments in the same publication. And then trying to track down which methods actually correspond to which experiments and which result is really, really hard. (…) Even tracking down what papers result from which grant is not that straight forward. |
| Saved for future publication | R01 | Q20 | | Manchmal kommt es tatsächlich zu dem Umstand, dass man sagt: „Nein, das passt nicht rein“, weil hier wird plötzlich etwas beleuchtet, an das man vorher gar nicht gedacht hat. Und dann kommt es halt in die nächste Publikation rein. Das heißt, was wir tatsächlich ab und zu haben, ist: Ja, wir haben vier Experimente in einem Tierversuchsantrag drin, nur zwei davon gehen gemeinsam in eine Publikation und die anderen würden jeweilig in eine andere reingehen. | Sometimes it happens that you say: ‘No, that doesn’t fit [into this paper] because suddenly something is flagged that you hadn’t even thought of before. So then it goes into the next publication. Sometimes we have four experiments within a *Tierversuchsantrag*, only two jointly go into a publication, and the others each go into a different one. |
| Saved for future publication | R03 | Q21 | | Wenn ich jetzt dieses negative Ergebnis in einem Low Impact Factor Journal veröffentlicht hätte, sofort nachdem die Promotion da ist, um einfach das negative Ergebnis auch zu publizieren, wie es ja erwünscht ist, hätte ich zwei Jahre später nicht die Möglichkeit gehabt, das als Kontrollregion mit dazu zu nehmen und dadurch die Studie so aufzuwerten, dass ich sie höher publizieren kann. | If I had published this result in a low impact factor journal straight after my doctorate, just to also publish the negative result, as is desirable, I wouldn’t have had the opportunity two years later to include that as a control and thereby upgrade the [new] study so that I can publish it better [in a more highly ranked journal]. |
|  | | | **CAUSES OF INCOMPLETE REPORTING** | |  |
| **Lack of incentives to report negative and null results** | | | | |  |
| Hard to publish high impact | R01 | Q22 | | Die Hauptfaktoren sind natürlich, dass es sehr, sehr schwierig ist, Negativergebnisse zu publizieren, dass viele Journals das nicht akzeptieren. | The main factors are of course that it is very, very difficult to publish negative results, because many journals do not accept that. |
| Hard to publish high impact | R02 | Q26 | | Wenn es ein einzelnes, negatives Ergebnis ist, und man hat 20 andere Paper zu veröffentlichen mit positiven Resultaten, ist es auch sehr wahrscheinlich, mit welchen man sich zuerst beschäftigen würde, weil sie einen größeren Impact meinetwegen versprechen und so weiter. (…)Gebe ich jetzt alles auf eine große Publikation, um dann sichtbar zu werden, oder – in Anführungsstrichen – verschwende ich auch Zeit für die Publikation von negativen Resultaten, die dann möglicherweise in vielen Jahrzehnten anderen Leuten zugutekommen? Das kann man nicht den jungen Nachwuchsleuten aufbürden. Aber letztendlich sind es die, die die Daten generieren und die dann auch viel zuarbeiten müssten. | If it’s a single, negative result, and you have another 20 papers to publish with positive results, then it is very likely which you will tackle first, because they promise a high impact and so on. (…) Do I aim at one big publication, to become visible, or do I – in inverted commas – “waste” time for the publication of negative results that possibly benefit other people in decades down the line? You can’t burden young ECRs with that. But at the end of the day, it’s them who have to generate the data and do the groundwork. |
| Hard to publish high impact | R03 | Q51 | | es gibt auch Arbeitsgruppen, die sagen: „Wenn das nicht Nature ist oder wenn das nicht mindestens Impact-Faktor 10 ist, dann publiziere ich erst gar nicht.“ (…) Aber die meisten, ist dann doch, dass man denkt „Ja, das sind Teilversuche, die man über Jahre dann hinterher in gebündelter Form dann einreicht“. | There are also working groups that say, ‘If it isn’t Nature or if it isn’t at least impact factor 10, then I’m not going to publish that.’ But most groups think, ‘those are partial experiments that you then submit in aggregated form years later’. |
| Hard to publish high impact | R04 | Q24 | | es war nicht das erste Mal, dass wir damit Probleme hatten. Dass wir Sachen veröffentlichen wollten, die man abgelehnt hat, weil es keine signifikanten Ergebnisse gab. | It wasn’t the first time that we had problems with that, that we wanted to publish things that were rejected because there were no significant results. |
| Hard to publish high impact | R05 | Q27 | | Und dass man das dann nur als Beschreibung und Beobachten publizieren kann, ist schwierig. Kann man, aber da muss man auch wissen, das ist für viele Wissenschaftler nicht wert, das zu publizieren. Da ist auch der Anspruch da, dass das einen gewissen Impact-Faktor hat, weil man sich sonst auch seinen Durchschnitt versaut. (…) und gerade wenn ich habilitiere z.B., muss ich ja einen gewissen Mittelwert, also meine Impact-Faktoren und Publikationen, da wird ja gemittelt | There’s also the standard that a publication should have a certain impact factor, because otherwise you mess up [“*versauen*”] your average (…) When I’m seeking tenure, for example, I need a certain average, my impact factor and publications get averaged. |
| Hard to publish high impact | R08 | Q25 | | Aber auf dem Weg dahin haben wir natürlich die ganzen anderen Dosierungen, die ganzen anderen Zeitpunkte, die anderen Infektionsarten mit dabei, die wir dann nachher nicht weiterverfolgen. Die tauchen in dem Paper wahrscheinlich nicht auf. Wenn Sie das machen, würde jeder Reviewer die raus streichen und sagen: „Das brauchen wir nicht, das gehört da nicht dazu.“ Aber die zu berichten, in einem Paper, das dürften Sie auch nicht, weil das wäre eine Doppeltpublikation, denn Sie haben ja die Gruppen daraus schon publiziert. | But on the way there of course we have all the other dosages, the other time points, the other types of infection, that we then don’t pursue. Those probably don’t appear in the paper. If you do that, every reviewer would delete them and say, “We don’t need those”. But you’re also not allowed to report them in a [different] paper because that would be double publication as you’re already published the same groups. |
| Fixation on p-values | R17 | Q23 | | Und aus einer Statistikersicht: P-Values haben gar keinen Wert.. (…) Und das fehlt bei der ganzen Publikationskultur. Und weil kein Sternchen dran ist, wird es nicht publiziert. Aber der Effekt, der auftritt, kann dennoch relevant sein. (…)Aber solange wir P-Values haben und solange dieser Publikationskult in diese Richtung geht, werden wir immer einen Publication Bias haben. | From a statistician’s perspective, p-values have absolutely no value. (…) And that is missing in the whole publication culture. Because there is no asterix next to it, it doesn’t get published. But the observed effect can nonetheless be relevant. (…) But as long as we have p-values and as long as the publication cult [“*Publikationskult*”] goes in this direction, we will always have publication bias. |
| Hard to publish replications | R05 | Q28 | | Ich habe vorher in der Immunologie gearbeitet und in der Impfstoffentwicklung. Und da geht es auf Zeit. (…) Nur wenn ich was Ähnliches oder Gleiches gefunden habe, dann wird es schwierig, das noch mal zu publizieren, weil das ist ja nicht mehr neu. Oder man kann es dann noch publizieren, aber nicht mehr in einem hochrangigen oder gleichrangigen Journal. | I’ve previously worked in immunology and vaccine development, and there it’s a race against time [“*da geht es auf Zeit*”]. (…) But when I’ve found something similar or identical, it gets difficult to publish it again because it is no longer new. Or you can publish it again, but no longer in a highly ranked or equally ranked journal. |
| When can null results be published ‘well’ | R02 | Q29 | | Was die Veröffentlichbarkeit von negativen Daten angeht, denke ich, hängt es dann sehr stark davon ab, wie konfrontativ so ein Ergebnis ist oder wie spektakulär, wenn meinetwegen über Jahrzehnte hin irgendeinen Mechanismus postuliert wurde und man zeigt jetzt an einem Tierexperiment, dass es das nicht ist. Da kann man sicherlich solche negativen Daten sehr hochkarätig publizieren (…) solange die Daten solide sind und gerade vielleicht auch mit neuen Technologien möglicherweise viel sauberer zu analysieren sind | Regarding the publication of negative data, it depends on how confrontational or spectacular a result is. If for example a certain mechanism was postulated for decades and now it is shown in an animal study that that is not the case, then you can surely publish such negative data very prominently. (…) As long as the data are solid and possibly even can be analysed much better with new technologies. |
| When can null results be published ‘well’ | R03 | Q31 | | wir haben auch festgestellt: Wenn wir gegen den Mainstream publizieren und vielleicht einfach mal zeigen, dass das nicht funktioniert hat, oder Gründe angeben, warum was nicht funktioniert hat, dass diese öfter zitiert werden als Studien, in denen nur positive Ergebnisse gezeigt werden. Weil andere Forscher da vielleicht endlich mal einen Grund dafür finden, warum ihre eigenen Sachen nicht klappen | We have noted that if we publish against the mainstream and just show that something has not worked, or give reasons why something has not worked, that these [publications] are cited more frequently as studies that only show positive results. Because other researchers maybe found a reason in there why their own attempts don’t work out. |
| **Pressures to deliver positive results** | | | | |  |
| Selective reporting | R03 | Q34 | | Aber ich bin ja auch Gutachter für viele Journals (…) Meistens wird vorne im Methodenteil das angegeben, was tatsächlich dann auch in den Ergebnissen publiziert wird, wo ich von meiner Erfahrung her sagen kann, das kann nicht sein, dass bei einem, ja, komplizierten Tiermodell, wo ganz, ganz viele Faktoren ineinandergreifen müssen, damit es dann final auch wirklich hundertprozentig passt/ Das kann nicht sein, dass alle Tiere immer funktionieren. | I’m a reviewer for many journals. (…) Usually it’s stated in the methodology section what then is also presented in the results, where from my experience I can say, that can’t be. That in a complex animal model where many, many factors have to align so that it all completely fits at the end. That can’t be, that all animals always function. |
| Selective reporting | R13 | Q32 | | [A] Also ich glaube schon, dass es [excluding outliers] eine Sache ist, die gemacht wird. Ja, also ich glaube schon. Also bei uns ist es keine gute wissenschaftliche Praxis, das weiß ja auch eigentlich jeder, dass man das so nicht machen sollte. Aber ich glaube schon, dass das auf jeden Fall gemacht wird. | I do believe that it [excluding outliers] is a thing that is done. I do believe so. With us, it’s not good scientific practice, and everyone here kind of [“*eigentlich*”] knows you shouldn’t do that. But I do believe that it’s definitely being done. |
| Selective reporting | R08 | Q33 | | Wir haben ja immer ungefähr gleich große Tiergruppen, sechs bis acht oder so, oder vielleicht auch mal zehn. Wenn dann plötzlich eine Gruppe zehn hat, dann kriegt man ein komisches Gefühl. Schlagen Sie mal Nature & Science auf, die größten Wissenschaftsjournale: Da haben Sie ganz viele Mausversuche, die genau drei Tiere in einer Grafik haben, das ist ein Klassiker von Nature & Science. Diese drei Tiergruppen sind super nah zusammen. Ich sage mal, das kann so sein, muss aber nicht.  [Q] Das sind die mit den schönsten Kurven.  [A] Genau, das sind die mit den wenigsten Tieren. Ich will niemandem etwas vorwerfen, aber ich glaube, das ist die häufigste Art von Fault, dass Tiere rausgenommen werden. (…) Ich stehe nicht hinter jedem Menschen, der pipettiert. Aber ich glaube, dass so was natürlich passiert. Die Leute sind unter Druck, die brauchen einen Job, die haben sonst nichts zu tun, – da muss man sich nichts vormachen. Und ich glaube, das kann dann auch zu falschen Menschenversuchen führen. | We always have roughly identical animal group sizes, six to eight or so, maybe sometimes ten. (…) Open Nature and Science, the largest scientific journals: There you have very many mouse experiments that have exactly three animals in a chart, that’s a classic of Nature and Science. These three animal groups are super close to each other. I’ll say, that may be so, but doesn’t have to be. (…) Those are those with the fewest animals. I don’t want to accuse anyone of anything, but I think that’s the most frequent kind of fault, that animals are excluded. (…) I don’t stand behind every person using a pipette. But I believe that of course things like that happen. People are under pressure, they need a job, else they don’t have anything to do – there’s no need to deceive ourselves. And I believe that that can also lead to wrong [“*falschen*”] human trials. |
| **Perceptions that some data do not add value** | | | | |  |
| Depends on context | R07 | Q52 | | Es gibt Sachen, die fallen hinten runter, da würde ich wirklich absolut sagen: Das ist so, da sehe ich überhaupt kein Problem. Und bei anderen wieder: Das macht mir Bauchschmerzen, dass es nirgendwo auftaucht. | There are things that fall between the cracks where I would really absolutely say: It’s like that, I see no problem at all. And with other things: It gives me stomach aches [“*gibt mir Bauchschmerzen*”] that it doesn’t appear anywhere. |
| Data ruined | R16 | Q35 | | es natürlich unerwartete Vorkommnisse geben und das sind die Dinge, wo ich dann sage, die möchte ich auch nicht als Negativdaten publiziert haben. Ich sage jetzt mal, es gab technische Probleme. Während der Studie ist die Klimaanlage ausgefallen und es waren draußen 30 Grad oder minus 15 Grad, sodass es eben unbeabsichtigte Einflüsse gab, die man auch bei bester Planung und bei besten Fähigkeiten nicht verhindern konnte. (…) Also fehlerhafte Daten sind ja keine Daten, die die Wissenschaft weiterbringen. | There can be unforeseen events and those are the things where I then say, I don’t want them published as negative data. During the study the air conditioning failed… so there were unintentional influences that the best planning and competence could not prevent. (…) So, erroneous [“*fehrlerhafte*”] data are not data that advance science. |
| Dropout pre-experiment | R01 | Q36 | | In unseren Käfigen dürfen Sie bis zu fünf Tiere gleichzeitig halten und Sie applizieren dem einen Tier die Zellen, den anderen Tieren auch, dann kommt es immer mal wieder vor, dass tatsächlich diese Tiere sich gegenseitig quasi attackieren und dementsprechend sich gegenseitig töten. Und die fallen dann natürlich ebenfalls raus. | It happens occasionally that animals attack each other [*within shared cages*] and therefore kill each other. And those drop out as well, of course. |
| Dropout pre-measurement | R11 | Q37 | | man erkennt es hinterher erst, wenn man den Versuch gemacht hat, die Tiere euthanasiert, obduziert, sich die Bioverteilung anschaut und dann sieht: „Das, was wir da appliziert haben, ist eigentlich da, wo es hinsollte, gar nicht angekommen.“ (…) Also wir haben im Prinzip kein Ergebnis, weil die Fragestellung gar nicht adressiert wurde. | You only realise it afterwards, once you’re done the experiment, euthanised and autopsied them, looked at the bio-distribution, and then see: “What we applied didn’t reach its intended destination.” (…) So basically we have no result, because the [research] question had not been addressed. |
| Dropout pre-measurement | R01 | Q38 | | Das heißt, bei 40 Prozent hat es nicht funktioniert, aus welchen Gründen auch immer. Das sind dann die biologischen Gründe, die wir nicht nachverfolgen, weil das nicht das Ziel ist. (…) das bringt nichts, wenn wir jetzt einem Tier, das nicht rekonstituiert wurde, dann halt humane Haut transplantieren und sagen: „Diese Haut wurde nicht abgestoßen.“ Sie wurde natürlich deshalb nicht abgestoßen, weil das Tier gar kein Immunsystem hatte und dementsprechend passt es nicht in irgendeine Gruppe. | So it didn’t work with 40%, for whatever reasons. Those are the biological reasons that we don’t pursue because that’s not the aim. (…) It’s futile if we transplant human skin onto an animal that hasn’t been reconstituted and say, “This skin was not rejected.” Of course it was not rejected, because the animal had no immune system whatsoever, and accordingly it does not fit into any group. |
| Experiment terminated early after tiny pilot group | R13 | Q39 | | Das heißt, selbst wenn wir einen Tierversuchsantrag haben mit fünf Gruppen, so und so viele Tiere, werden vielleicht am Ende nur drei Gruppen nachverfolgt und der Rest wird liegengelassen, weil man merkt, das ist nicht praktikabel oder weiß ich nicht. (…) Das sind ja keine echten Statistiken, sag ich mal. Man sieht einen Trend dann, bei einer geringen Tierzahl und hat gemerkt „Es geht nicht“, oder man hat gemerkt: Die andere Gruppe ist sinnvoller. (…) es ist dann die Frage, wie aussagekräftig das vielleicht ist.  [Q] [Potential value for meta-analyses?]  [A] Also ich glaube, da gibt es zu wenig overlap. (…) Ich glaube, um exakt den gleichen Versuch irgendwo noch mal zu finden/ Ist fast unmöglich. Also, dass irgendeine Variable immer sich ändert, in den Versuchen. | If we have a *Tierversuchsantrag* with five groups, so and so many animals, at the end only three groups are pursued and the rest get dropped because you notice that this is not practicable or whatever. (…) Those are not real statistics, I say. You see a trend, with a low number of animals, and notice “that doesn’t work,” or notice that the other group is more sensible. (…) The question is then how much sense that makes [to publish those data]. (…) I think there is too little overlap [for such data to be useful for meta-analysis. (…) I think it’s nearly impossible to find the same experiment elsewhere, some variable always changes in experiments. |
| **Commercial pressures** | | | | |  |
| Commercial influence | R10 | Q42 | | [Example] looking at something in terms of the specific intervention and the impact that that medical intervention is having on a particular group of animals over a period of time. So that would be the closest equivalent [to trials]. The difficult is that we have with that and actually the commercial entities really recognize this is that they are the people who are least likely to share their results in a public forum because they have issues of commercial sensitivity, confidentiality and competitiveness which by law bind them and make it difficult for them to share information even if they wanted to. | Commercial entities… are least likely to share their results in a public forum because they have issues of commercial sensitivity, confidentiality and competitiveness which by law bind them and make it difficult for them to share information even if they wanted to. |
| Commercial influence | R14 | Q41 | | Das kommt halt immer ein bisschen darauf an, was für Verträge man mit denen abschließt. Wir sind ja als Uni in der glücklichen Lage, dass wir meistens oder eigentlich ausschließlich Forschungs- und Entwicklungsverträge haben. Und da steht bei uns in den Statuten, ich mach die Verträge teilweise auch mit, drin, dass es klar geregelt ist, dass wir die Informationen für die Veröffentlichung, dass die quasi in unserer Hand sind. Sie haben die Möglichkeit, ich glaube, in einem Rahmen von vier bis sechs Wochen zu antworten, also dass man denen das vorher schickt, das Manuskript, und sich dazu zu äußern… Also die können dann schon mit einem diskutieren, aber im Endeffekt ist vertraglich festgelegt, dass wir die Sachen für die Veröffentlichung so nutzen dürfen.  Wenn man komplett im Sinne von Auftragsforschung arbeitet… da ist das natürlich was anderes. Da ist das: „Ich hab das eingekauft und die Studie hat nicht das Ergebnis geführt, dann wird die Studie eingefroren und dann wird die Studie an einem anderen Standpunkt eventuell noch mal gemacht.“ Da ändern sich dann zwei, drei Komponenten, wo man dann sagen muss „Na ja, eigentlich bleibt es trotzdem das Gleiche“, aber dann macht man die Studie halt drei-, vier-, fünfmal bei einem anderen CRO, bis das rauskommt, was man haben will. (…)  Gerade bei den Auftragsforschungsgeschichten habe ich oft den Eindruck, dass man sich quasi versucht, seine Hypothese zu erkaufen. So, und wenn die nicht zielführend ist, dann wird es mehr oder weniger lieber unter den Teppich gekehrt, als dann irgendwie in Verbindung gebracht zu werden mit einem Produkt (…) Also die Industrieseite, die möchte die Negativdinge nicht publiziert haben. | If you work completely within contract research [outside a university] it’s different. There it’s: “I bought that and the study didn’t show that result, so it gets put on ice and possibly the study gets done again at a different site.” (…) Then the study is done three, four, five times with a different CRO until you get the result that you want. (…) I often have the impression there’s an attempt to buy the hypothesis. And when it isn’t expedient, then it gets swept beneath the carpet rather than somehow being brought into connection with a product. (…) So from the side of industry, they don’t want negative things to be published. |
| **Reputational concerns** | | | | |  |
| May suggest study was flawed | R01 | Q43 | | War das eine wohldurchdachte Hypothese oder war das von vornherein eine Hypothese, die überhaupt nicht funktionieren kann? (…) wenn man sich im Vorfeld mit der Literatur nicht beschäftigt hat und plötzlich eine Hypothese aufstellt, die überhaupt nicht funktionieren kann, egal was man macht, dann ist das für mich kein wissenschaftlicher Erfolg | Was that a thought through hypothesis, or was that from the outset a hypothesis that cannot work at all? (…) If you haven’t engaged with the literature up front and suddenly formulate a hypothesis that cannot work at all, no matter what you do, then for me that’s not a scientific success. |
| Drop-out rates can reflect on competence | R11 | Q44 | | Aber ich glaube, wenn man das öffentlich machen wollte, dann ist es durchaus sinnvoll, das anonym zu halten, weil das sonst ein ganz starker Anreiz wäre, dass man das auf gar keinen Fall publik machen sollte, weil man sich dadurch ja wirklich extrem angreifbar macht. Wenn es heißt, irgendwie: „Guck mal, in dem Labor läuft immer alles schief, die brauchen viel mehr Mäuse als wir, für das gleiche Modell“ oder so, „weil sie irgendwelche Fehler machen“. Es wäre natürlich wünschenswert, dass es so eine Fehlerkultur gibt. | I believe that if you want to make that public, then it makes sense to keep it anonymous. Because otherwise there would be a very strong incentive not to make that public under any circumstances, because you would make yourself extremely vulnerable to attacks. If the word is: “Look, in that laboratory everything always goes wrong, they need many more mice than we do, for the same model.” Of course such a culture of openness about failures [“*Fehlercultur*”] would be desirable. |
| Drop-out rates can reflect on competence | R15 | Q45 | | I think most of the reason that we picked up [for not using ARRIVE] was more you know they basically people who are actually reluctant to give too much information because they were worried that it would reveal flaws in the studies (…)people don’t want to say that their surgery is only effective in 50 percent of the time (…) they want to give this impression that everything works all the time but science is messy | I think most of the reason that we picked up [for not using ARRIVE] was… basically people who are actually reluctant to give too much information because they were worried that it would reveal flaws in the studies (…) People don’t want to say that their surgery is only effective 50 percent of the time (…) They want to give this impression that everything works all the time, but science is messy. |
| **Socio-political and regulatory pressures** | | | | |  |
| Stigma and external pressure | R06 | Q49 | | Ich denke, man muss einfach weggehen von diesem: Ich werde kontrolliert oder sogar bestraft. Es ist teilweise erschreckend. Wenn man versucht, die Regeln zu befolgen, hat man eigentlich mehr Angst, dass mit dem Finger auf einen gezeigt wird, weil plötzlich gibt es was zu kontrollieren. Wenn ich aber nichts dokumentiere, laufe ich teils weniger Gefahr. | I think we need to get away from this, ‘I am being controlled or even punished’. Sometimes it’s scary [“*erschreckend*”]. If you try to follow the rules, you actually are more afraid that someone could point the finger at you, because there is suddenly something to control. But if I don’t document anything, I run less danger. |
| Stigma and external pressure | R07 | Q46 | | weil es das Stigma Tierforschung an sich ist. (…) Dass man sich vermutlich unbewusst, aufgrund der Gesellschaft drumherum, bei Tierversuchen sehr zurückhält, dann natürlich: „Oh Gott, ich will ja nicht als schlecht dastehen.“ Dann dazu kommt, nur das Positive zu erzählen und nicht, was man im Vorfeld alles hatte. | It’s the stigma of animal research in itself. (…) That you, presumably unconsciously, because of wider society, are very reticent about animal research, and then: “Oh God, I don’t want to be seen as bad.” Which leads to only wanting to talk about positive things, and not everything that happened in the run-up. |
| Stigma and external pressure | R11 | Q47 | | Wenn man Tierversuche durchführt, muss man dafür ja einen guten Grund haben, und wenn der Grund nicht gegeben ist, weil die Versuche nicht klappen, hat man genau dieses Problem mit dem Stigma. (…) Wir haben halt einfach eine Situation, in der es noch erheblichen Druck gibt (…) durch behördliche Kontrolle und vor allem eben auch den öffentlichen Druck. (…) In der Tierforschung ist es ja schon auch so, dass man den Eindruck hat, man stehe immer mit einem Fuß schon im Gefängnis. (…) Da ist man ja oft schon direkt im strafrechtlich relevanten Bereich. Und dieses Gefühl, […] das ist wie ein Damoklesschwert, über der ganzen Forschung. | If you run animal experiments you need a good reason, and if that reason is invalidated because the experiments do not work out, then you have exactly this problem with the stigma. (…) We just have a situation in which there is considerable pressure… through regulatory control and especially also public pressure. (…) In animal research you have the impression of always already having one foot in prison. (…) You’re often already in the sphere of criminal law. And this feeling, […]that is like a Sword of Damocles, over the entire research. |
| Stigma and external pressure | R11 | Q48 | | [Q] Ist es in der Tierforschung so, dass es da tatsächlich Gruppen gibt, die systematisch nach Talking Points suchen würden in der Literatur, wenn man da Fehlschläge berichten würde? Das würde dann auf einem öffentlichen Radar aufschlagen?  [A] ganz eindeutig ja (…) wir wissen von mehreren Fällen, wo das passiert ist und genau das dazu geführt hat, dass bestimmte Kampagnen initiiert wurden. (…) Also das ist definitiv der Fall, dass die professionell organisierten Organisationen in dem Bereich wissen, wie man Studien liest und natürlich daraus auch zum Teil sehr Cherry Picking und sehr, ja, missinterpretierende Fazite ziehen. | [QUESTION] In animal research is it really the case that there are groups that would systematically look for talking points in the literature? If you reported failures, that would appear on the public radar?  [ANSWER] Definitely. (…) We know of several cases where that happened and directly led to the initiation of certain campaigns. (…) It’s definitely the case that professionally organised campaigns in the area know how to read studies and cherry pick and draw very misinterpreting conclusions. |
